# Supplementary material for: Antifungal Activity of Phyllospheric Bacteria Isolated from Coffea arabica against Hemileia vastatrix
Source: Microorganisms. 2024 Mar 14;12(3):582. doi: 10.3390/microorganisms12030582 (PMC10976141; doi:10.3390/microorganisms12030582)
Supplement: Supplementary file 1 [file microorganisms-12-00582-s001.zip › microorganisms-2784712-supplementary.pdf]

Suplementary

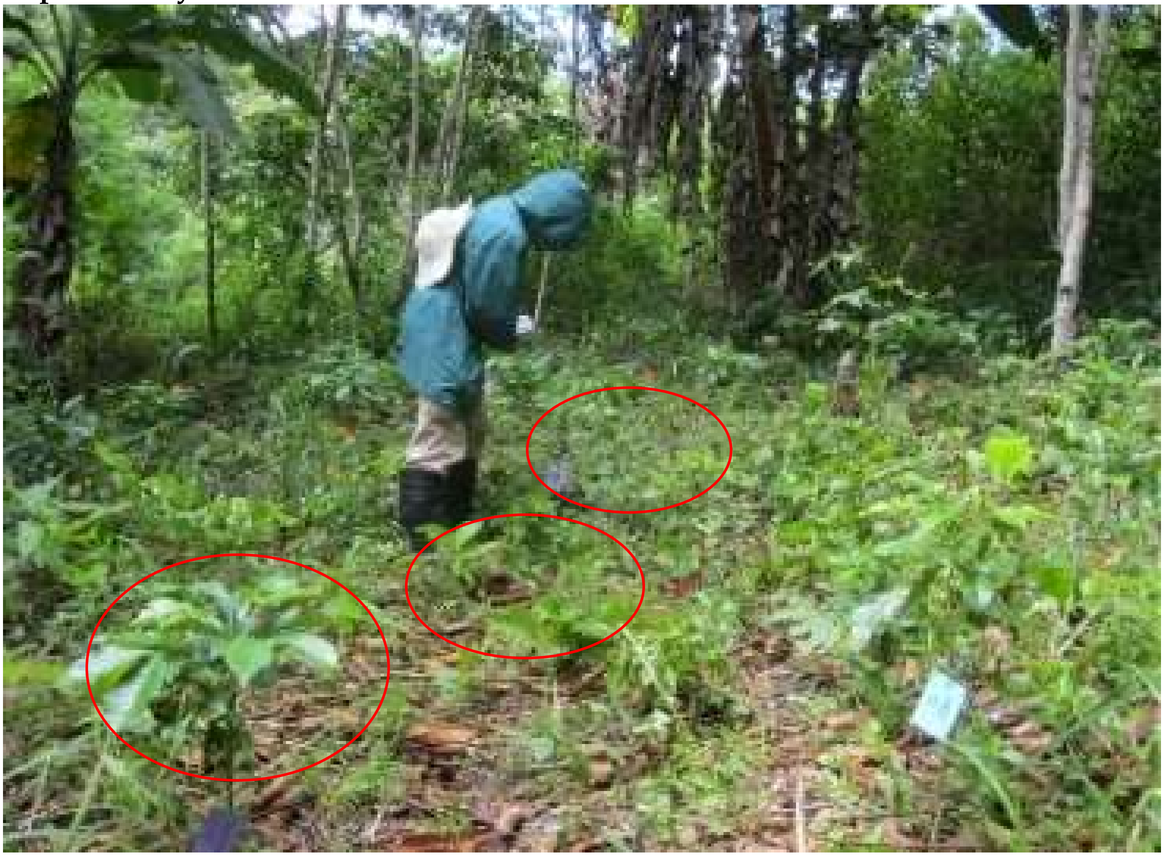

Fig S1. Foliar spray inoculation on coffee field assay. Some coffee seedlings are circled in red.
